# A Comparative Study of the Mechanical Properties of FDM 3D Prints Made of PLA and Carbon Fiber-Reinforced PLA for Thin-Walled Applications

**DOI:** 10.3390/ma14227062

**Published:** 2021-11-21

**Authors:** Jerzy Bochnia, Malgorzata Blasiak, Tomasz Kozior

**Affiliations:** Faculty of Mechatronics and Mechanical Engineering, Kielce University of Technology, 25-314 Kielce, Poland; jbochnia@tu.kielce.pl (J.B.); mblasiak@tu.kielce.pl (M.B.)

**Keywords:** 3D printing, FDM, carbon fibers, polymers

## Abstract

This study focused on the analysis of the mechanical properties of thin-walled specimens fabricated by fused deposition modelling (FDM). Two materials were considered, i.e., polylactide (PLA) and polylactide with carbon fiber (PLA-CF). The article describes how the specimens with different thicknesses and printing orientations were designed, printed, measured to assess their geometric and dimensional accuracy, subjected to tensile testing, and examined using scanning electron microscopy. The data provided here can be used for further research aimed at improving filament deposition and modifying the base material by combining it with different components, for example carbon fiber. The investigations revealed that the properties of thin-walled elements produced by FDM varied significantly depending on the thickness. So far, this problem has not been investigated extensively. Research by analyzing the key parameter, which is the direction of printing that is important for thin-walled models, provides a lot of new information for designers and technologists and opens the way to further extended scientific research in the field of the strength analysis of thin-walled models produced by 3D printing, which is very applicable to structure optimization in the era of the industrial revolution 4.0 and progress in the LEAN manufacturing process.

## 1. Introduction

Additive manufacturing (AM) has received much attention over the last few years. AM technologies are becoming increasingly popular as an attractive alternative to conventional manufacturing, especially CNC machining, injection molding, and casting [1], because they are suitable for short series production, rapid prototyping, and rapid manufacturing. Three-dimensional (3D) printing is particularly important with regard to the Fourth Industrial Revolution (IR 4.0). The most common additive manufacturing methods are selective laser sintering (SLS) [2,3,4] and fused deposition modeling (FDM), with the latter using thermoplastic materials. Elements made in this way do not require any surface engineering operations, e.g., heat treatment, chemical surface modification [5], or machining [6]. FDM has numerous industrial applications; it is specifically suitable for rapid manufacturing of customized products. Printing by FDM has many benefits, with the most important being: high strength of prints, a wide range of materials to work with, low cost per part, and the possibility to print prototypes of mechanisms to check their functionality [7,8]. What is more, the FDM technology can be applied to create elements with no geometric limitations, and there is also no need for drilling or other machining operations required in most 3D printing technologies involving metals [9]. Finally, it is possible to eliminate or reduce residual stress. The key characteristic feature of FDM, also known as fused filament fabrication or filament freeform fabrication (FFF), is material extrusion process, which involves feeding plastic material through a heated print head. Models are built by depositing molten thermoplastic material layer by layer. FDM-made elements may require additional support structures. Initially, the method was used to create cheap prototypes and concept models. Today, it is employed to print high-quality functional prototypes and concept models. An important advantage is the possibility to design models as cellular structures, e.g., honeycomb cellular structures, which reinforce the element but reduce its weight. This is crucial when production or assembly tools produced in this way are handled by human workers [10]. The most common thermoplastic materials used for FDM 3D printing are: ABS (acrylonitrile butadiene styrene), ASA (acrylonitrile styrene acrylate), PC (polycarbonates), and PLA (polylactic acid). Elements made of these plastics can be as strong as their metal counterparts, but they weigh less [11]. With the use of special filaments, it is possible to achieve comparable strength, impact resistance, and stiffness at lower material density. Some 3D printing plastics have sufficient properties to produce components that are traditionally made of metals. Except for high strength, they are resistant to various chemicals, including lubricants; they can be certified as non-combustible or biocompatible. In rapid manufacturing of customized polymer elements, there is no need to prepare a costly mold, as is required in casting or injection molding. However, this may have some drawbacks. For example, high anisotropy of 3D-printed objects with regard to their internal structure [12,13] makes it difficult to control their print time or their mechanical properties and surface texture, e.g., their roughness or waviness, depending on the technology used [14,15,16]. The mechanical properties of FDM-printed elements made of pure thermoplastic materials can be enhanced by using, for instance, carbon fiber or fiberglass reinforcement [17,18] and/or special additives, whose role is to improve the mechanical, electrical, and/or magnetic properties [19]. Composites reinforced with carbon fiber have perfect mechanical properties; they are stiffer and more impact and fatigue resistant. Because of the low density of carbon fibers, all these properties can be achieved at a low mass of the product. The use of carbon fiber or fiberglass in selective laser sintering (SLS) contributes to higher strength of elements made [20,21]. Valvez et al. [22] propose a review of the literature on the testing of FDM prints. The study focused on PLA reinforced with fiberglass or carbon fiber, which was done for the purpose of improving the mechanical properties of lower mass models and thin walled models. Reinforcing composites with fiberglass or carbon fiber aims to enhance the mechanical properties, especially the modulus of elasticity under tension [23]. The relative content of carbon fibers has a considerable effect on the mechanical behavior of composites under tension. An increase in the layer height and the extrusion width affects the proportion of carbon fibers in composites; it is responsible for a decrease in the mechanical properties when under tension [24]. A comparative study of the specimens made of 3D printed composites with and without carbon fiber is discussed in [25]. Adding carbon fibers to partly melted PLA particles improves the tensile and flexural strength of prints. The behavior of additively manufactured polymer elements under tension differs from that of, for example, steel elements manufactured using traditional methods [26]. The strength, surface characteristics, and the geometric and dimensional accuracy of PLA and PLA-graphene prints are discussed in [27]. After reinforcement with graphene, the strength of this material doubles; the modulus of elasticity also increases. Moreover, additives added to PLA may be responsible for higher surface roughness. The research presented in [28] deals with the testing of FDM- and CFF-printed composite models with a standard thickness of more than 2 mm taking no account of the printing direction. In another study [29], FDM printers were modified to enable the use of both pure PLA and PLA reinforced with carbon fiber (PLA-CF). The tensile strength and flexural strength tests revealed that carbon fiber reinforcement increased the strength of prints by 30–50%. Two methods of fabrication of ABS composites reinforced with carbon fibers, i.e., compression molding and FDM, were compared in [30]. The experimental data showed that the values of tensile strength obtained for either type of element was low. After adding carbon fibers, both types of print had higher tensile strength and higher elasticity modulus. In the FDM technology, where the height of a single layer may reach 0.2 mm, the surface quality should be analyzed with regard to surface waviness, due to greater irregularities or higher wavelengths (waviness) on the surface [31]. Surface waviness is significant also because it affects the vibration of mechanical elements [32,33,34]. To improve the strength and fatigue resistance of polymer composites, carbon fiber was placed in between the layers of the 3D-printed polymer [35]. The investigations showed that the larger the number of carbon fiber layers, the larger the size of voids, which had a negative impact on the tensile strength of prints. Poor bonding of PLA with carbon fiber may have a substantial effect on the mechanical properties of elements, surface adhesion, as well as tensile or flexural strength; this, however, can be improved by applying methylene dichloride and PLA granules [10,25]. The literature in this area covers both experimental studies and theoretical considerations [36,37], all aiming to find an optimal combination of materials characterized by higher adhesion. The printing temperature and speed may have influence on the bonding at the carbon fiber/matrix interface [24]. Diffusion may occur when materials are coated with the same or a similar polymer; as a result, their adhesion increases [38,39]. 

This article analyzes tensile test results for two materials, i.e., pure PLA and PLA reinforced with carbon fiber. The SEM examinations explain the mechanisms affecting the strength of the two types of prints.

## 2. Materials and Methods

### 2.1. Materials

The experiments were conducted for specimens made of two polymers. One was polylactide (PLA), with a trade name EASY PLA, produced by Fiberlogy, Brzezie, Poland, and the other was a composite, consisting of polylactide and carbon fiber (PLA-CF) sold under the trade name of CarbonFil, by Formfutura from the Nijmegen, Netherlands. The mechanical properties of the materials used to build the tensile specimens are given in Table 1. Both materials were provided in the form of filament wound on a spool 1.75 mm in diameter.

### 2.2. Methods

Each 3D printing technology uses specific equipment and a specific process. They all, however, have some limitations. To choose a proper 3D printing method, we just need to answer some basic questions, i.e., how thick or thin, how big or small, and how precise the elements printed must be. Another problem to be dealt with is their physical properties, particularly mechanical and metrological properties. FDM is one of the most popular technologies with a large variety of applications. Since composites have recently seen an increase in popularity, extensive testing is necessary to determine the mechanical properties of such 3D models. The material is fed in the form of a filament from a spool to the heated nozzle by mechanical pressure from rollers. The temperature in the nozzle is regulated by the printing machine control system. Once the melting point is reached, the material in the filament form is first placed on the printer’s build plate and then as a successive layer on top of the previous layer, adhering to it mainly under the action of adhesive forces. In this way, the object printed has the same geometry as the model created using 3D CAD software. Its mechanical properties are slightly different from those of the material used for printing, i.e., a polymer filament. The properties of 3D prints are largely dependent not only on the type of printing material or the possibilities of the printing machine but also on the process or rather the control of the process parameters. In FDM, the key parameters involved in the printing process include printing direction, layer height, extruder temperature, ambient operating temperature, temperature of the movable build plate, temperature in the build chamber, deposition rate, nozzle diameter, and infill density. As 3D printing is a layer by layer fabrication process, properly selected parameters guarantee that the print has specific mechanical properties, which are largely dependent on the printing direction.

One of the aims of this study was to analyze the effects of the printing direction on the mechanical properties of thin-walled composite elements. There has been some research in this field however, it has focused on solid objects. From the above description, it is clear that the 3D printing process may have a significant effect on the mechanical properties of elements. The major forces responsible for the proper bonding of a polymer filament extruded onto the layer beneath it are adhesive and cohesive in character. Depending on the positioning or orientation of the model on the build plate, either type of the forces may predominate. The way filaments are arranged is also vital; there are differences between thin-walled elements and large solid elements with varying infill density.

The method described in this article allows thin-walled objects to be 3D printed in different orientations. Tensile tests were conducted for specimens differing in thickness and printing orientation to determine the tensile strength and the elasticity modulus. Currently, we can observe dynamic activities for standardization of 3D printing technologies and it seems, as the experimental data reveal, that 3D prints should be divided into those with a fully solid structure and those with thin walls; the latter may have a thickness of walls of less than 2 mm. So far such 3D samples have not been analyzed.

### 2.3. Preparation of the Fused Deposition Modelling (FDM) Specimens

The shape of the test specimens was designed in accordance with the ISO 527 standard. The exception is one dimension, i.e., the thickness of the specimens (1.0 mm, 1.4 mm and 1.8 mm), the impact of which was analyzed as part of further tests and compared with the reference specimen in fully compliant with ISO 527 with a thickness of 4 mm. The solid models of the specimens were created in SOLIDWORKS software (Dassault Systèmes SolidWorks Corp., Waltham, MA, USA) and saved as digital .*stl* files. The triangulation parameters used in the export options were as follows: resolution—adjusted; linear deviation—a tolerance of 0.016 mm; angle tolerance of 1. It is important that the values of the triangulation parameters should not be too low otherwise it will not be possible to create rounded objects. They should not be too high, either because such *.stl* files are too large for the embedded machine software to analyze.

**Figure 1:**
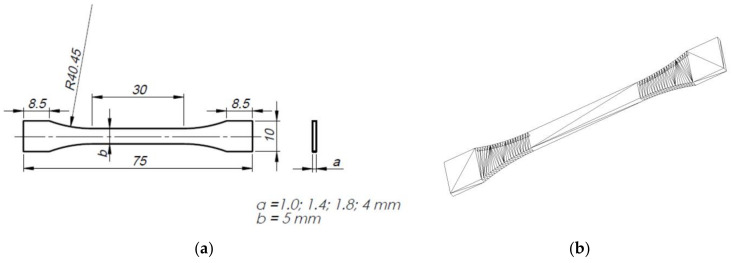
Tensile specimen, (**a**) 2D diagram with dimensions, (**b**) 3D sketch with a grid converted to triangles saved an .*stl* file.

Figure 1 shows a 2D diagram of a single specimen with dimensions and a 3D sketch with the grid after triangulation saved as an .*stl*-type file.

**Figure 2:**
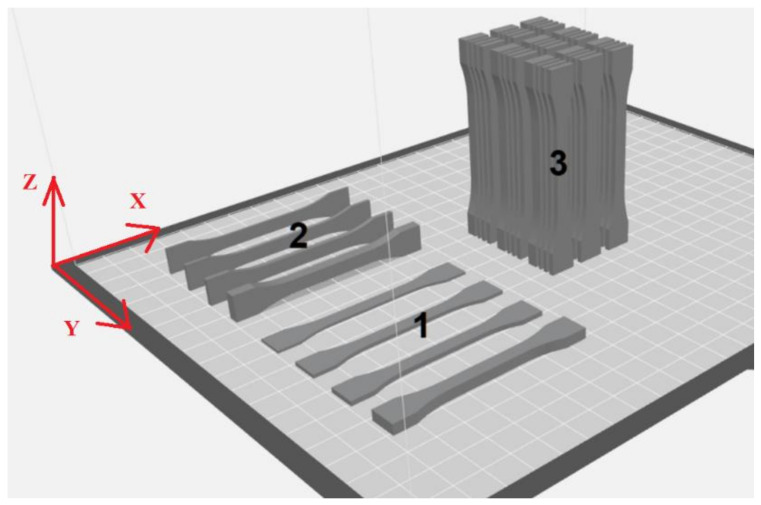
Arrangement of models on the build plate of the MakerBot printing machine; orientation along: 1—the *x* axis, 2—the *y* axis, and 3—the *z* axis.

The 3D model files saved in the .*stl* format were positioned on a virtual build plate of the MakerBot Replicator (5th Generation) printing machine (Brooklyn, NY, USA) in three orientations, as shown in Figure 2. This software automatically carries out the process of slicing the models into layers. The specimens oriented along the *x* axis had the largest flat surface on the build plate. The specimens oriented along the *y* axis had the side touching the build plate. The specimens oriented vertically along the *z* axis were placed close to one another to ensure stability in printing and prevent models from collapsing.

The printing parameters for both PLA and PLA-CF (mostly recommended by material producer and 3D printer manufacturer) used in the experiments were:

✓ Layer height: 0.2 mm;
✓ Infill density: 95% (maximum possible value in 3D printer software);
✓ extruder temperature: 230 °C for PLA and 250 °C for PLA-CF;
✓ nozzle diameter: 0.4 mm.

**Figure 3:**
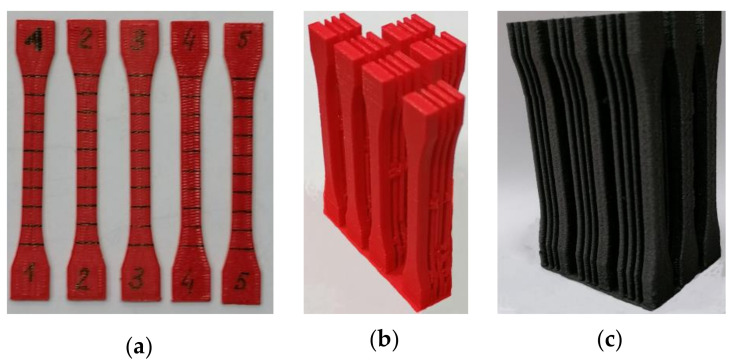
Examples of prints (**a**) marked PLA specimens (orientation along the *x* axis); (**b**) multiple PLA specimens made in one print (orientation along the *z* axis); (**c**) multiple PLA-CF specimens made in one print (orientation along the *z* axis).

The selected technological parameters affect the obtained mechanical properties and when other parameters are used, such as nozzle diameter, temperature, printing speed, cooling speed, the mechanical properties of the models produced may be slightly different. However, the mechanism of the influence of the print direction and thickness of the built models on the mechanical properties, regardless of the type of 3D printer in FDM technology, should be retained, which allows the presented test results to be related to different types of FDM printer. Examples of prints are shown in Figure 3.

After the printing was completed, the support material was removed from the build plate, the models were cleaned by removing the remaining filaments and measured to check their geometry, and finally the tensile tests were conducted. There were five specimens representing each type for the purpose of statistical calculations.

## 3. Results

### 3.1. Metrology

The thickness and width of each specimen built for the static tensile strength tests were measured along the gage length at three points, at the beginning, middle and at the end. Then, the average thickness *ā* and the average width $\bar{b}$ were calculated for both materials, i.e., PLA and PLA-CF. The results are provided in Table 2 and Table 3, respectively. 

The measurements were performed by means of a micrometer with an accuracy of 0.01 mm. The specimens were marked according to the nominal thickness, the specimen number in a series, and the printing direction (the No. column in the tables). For example, a specimen marked as 1.4 2X is a specimen with a nominal thickness of 1.4 mm, second in a series, oriented in the X direction on the build plate (Figure 1b). The PLA-CF specimens were additionally marked ‘C’; thus, a specimen made of PLA-CF with a thickness of 1.4 mm, second in a series, printed in the X-direction, was marked as 1.4C 2X. The nominal thickness and other nominal dimensions are values assumed at the design stage in the CAD software converted into an .*stl* file to ensure communication with the printer software. The actual dimensions, i.e., the dimensions of the prints, differ from the nominal dimensions.

### 3.2. Tensile Tests

The static tensile strength tests were performed at a crosshead speed of 1 mm/min using an Inspekt mini 3kN universal testing machine produced by Hegewald and Peschke MPT GmbH. 

The ultimate tensile strength *R_m_* was calculated by the embedded machine software according to the following formula:

(1) $$R_{m}=\frac{F_{m}}{\bar{a}\bar{b}},$$

where: *F_m_*—maximum load, *ā*—average measured thickness of the specimen, $\bar{b}$—average measured width of the specimen.

The average values of the specimen width and thickness (Table 2 and Table 3, respectively) were transferred to the LabMaster software database for each specimen individually so that the data could be plotted as a stress and strain curve and *R_m_* could be calculated. Such programs use nominal values of the specimen dimensions, the same for the whole series; the values of the dimensional deviations are taken into consideration when measurement errors are estimated. In the case of thin-walled specimens, this approach would provide distorted results for the whole experiment.

**Figure 4:**
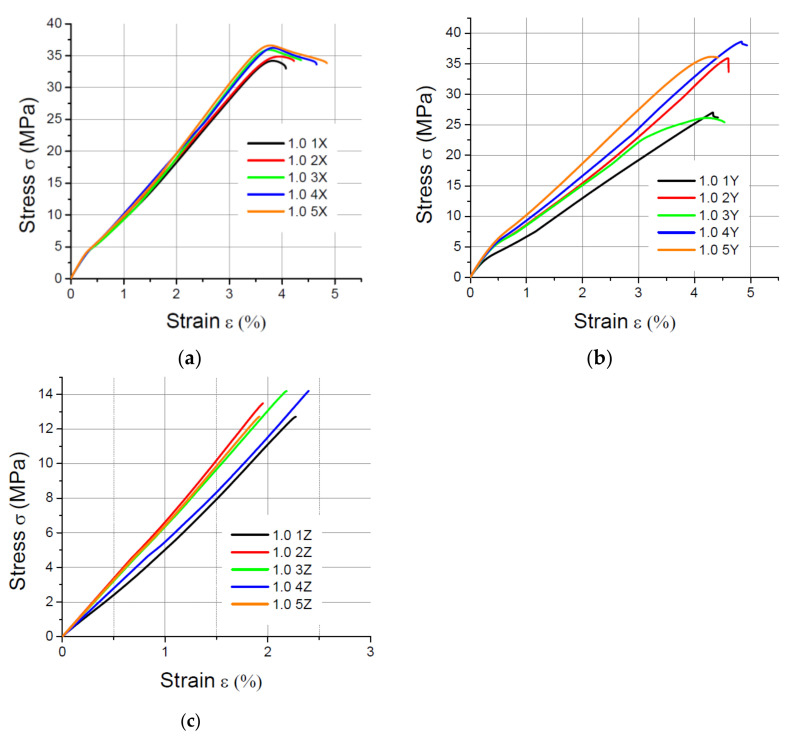
Tensile test results for 1 mm thick specimens made of PLA printed in (**a**) the X orientation; (**b**) the Y orientation; (**c**) the Z orientation.

**Figure 5:**
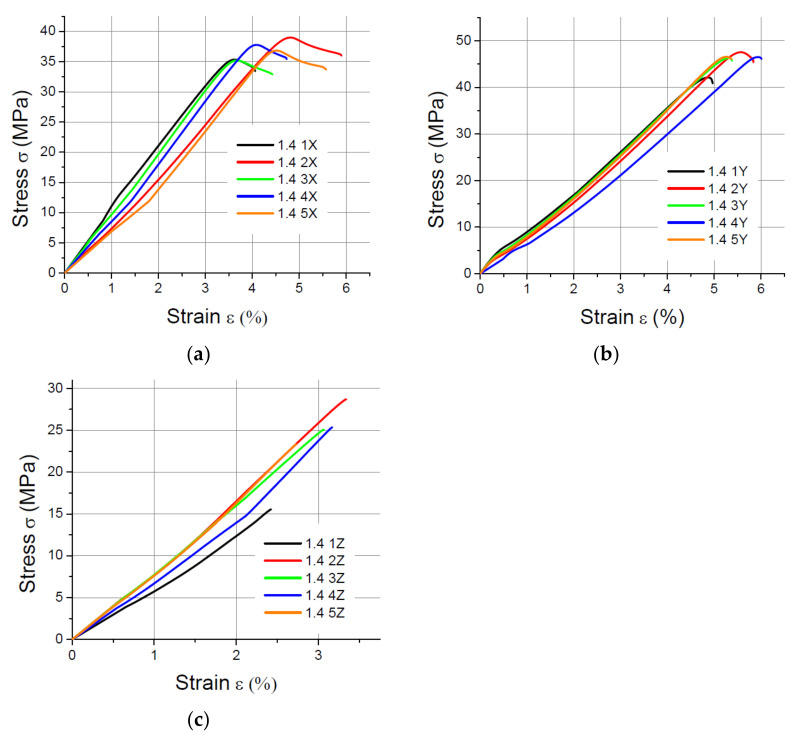
Tensile test results for 1.4 mm thick specimens made of PLA printed in (**a**) the X orientation; (**b**) the Y orientation; (**c**) the Z orientation.

**Figure 6:**
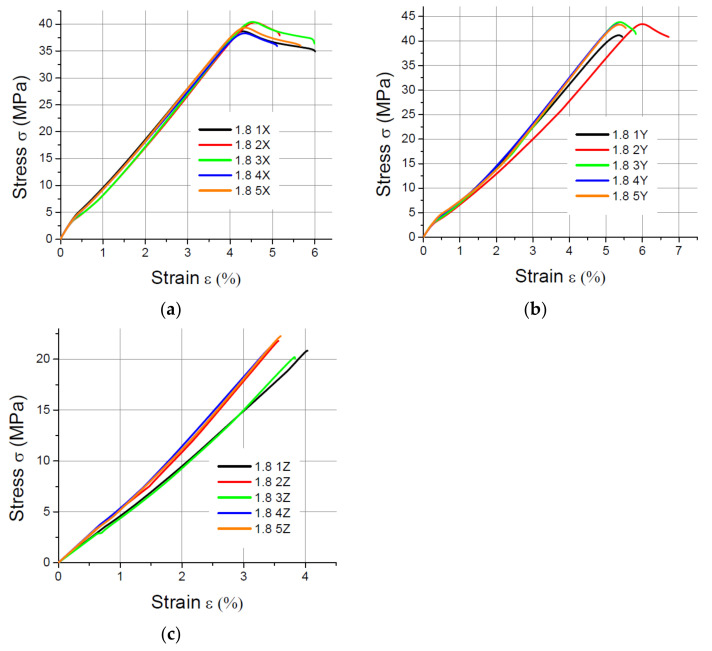
Tensile test results for 1.8 mm thick specimens made of PLA printed in (**a**) the X orientation; (**b**) the Y orientation; (**c**) the Z orientation.

**Figure 7:**
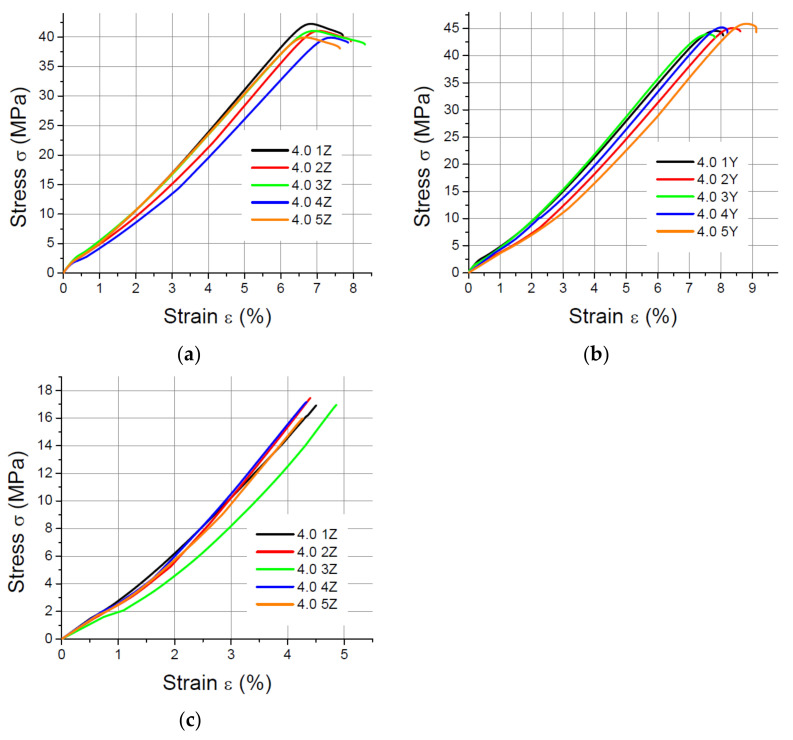
Tensile test results for 4 mm thick specimens made of PLA printed in (**a**) the X orientation; (**b**) the Y orientation; (**c**) the Z orientation.

The values of the elasticity modulus E were calculated automatically by LabMaster using the regression method for the prints oriented in the X and Y directions with deformations *ε* ranging from 0.2% to 2%, which guarantees the same straight measuring distance for all samples. For the specimens built in the Z direction, the elasticity modulus was determined also by means of the regression method, but it was assumed that the deformations *ε* varied between 0.2% and 0.8% because of the smaller elongation of the specimens printed vertically. The performed tensile tests showed that in the case of the printout in the “*z*” axis orientation, the unit strain is much smaller (in some cases the sample break occurred at a strain less than 2%) than for the *x* and *y* axis orientation, hence the adoption of two ranges of unit strains (strain). The results of the static tensile tests for the PLA specimens are shown in Figure 4, Figure 5, Figure 6 and Figure 7.

**Figure 8:**
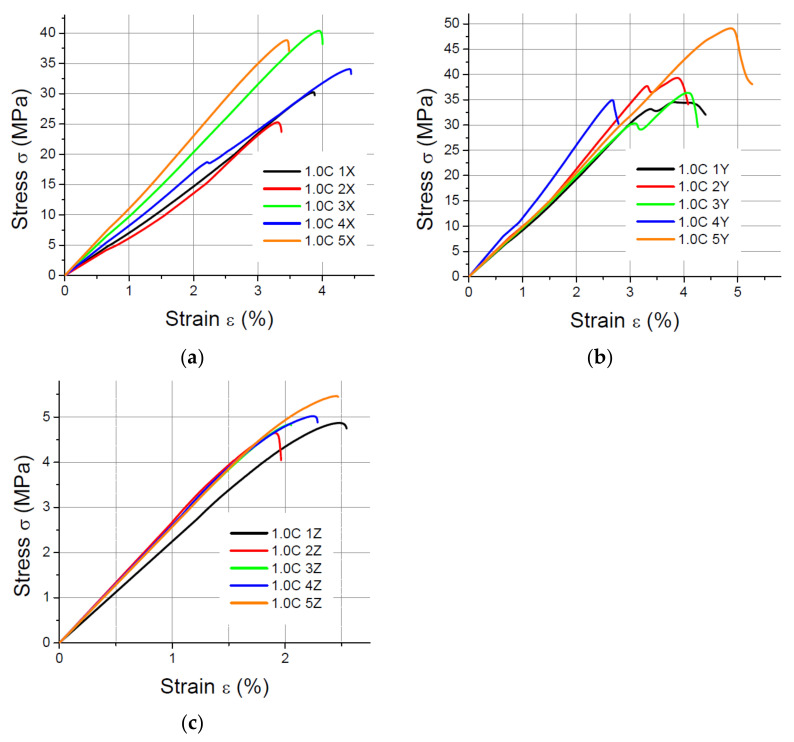
Tensile test results for 1 mm thick specimens made of PLA-CF printed in (**a**) the X orientation; (**b**) the Y orientation; (**c**) the Z orientation.

**Figure 9:**
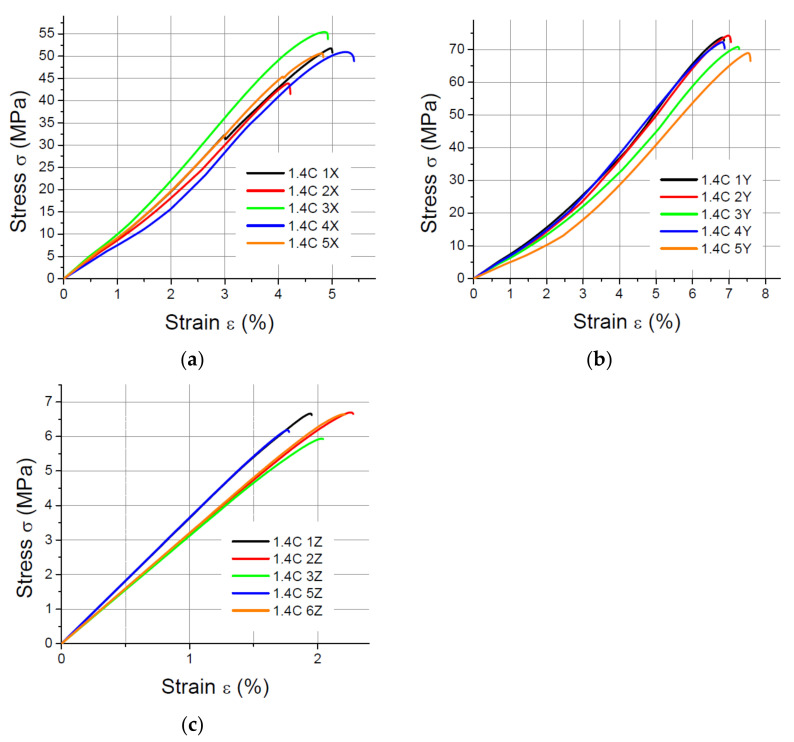
Tensile test results for 1.4 mm thick specimens made of PLA-CF printed in (**a**) the X orientation; (**b**) the Y orientation; (**c**) the Z orientation.

**Figure 10:**
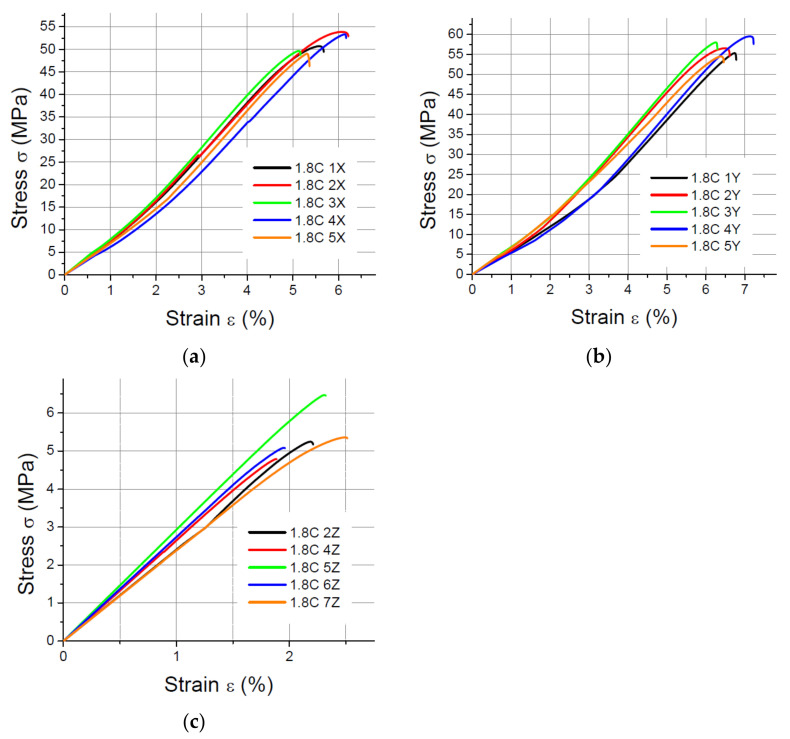
Tensile test results for 1.8 mm thick specimens made of PLA-CF printed in (**a**) the X orientation; (**b**) the Y orientation; (**c**) the Z orientation.

**Figure 11:**
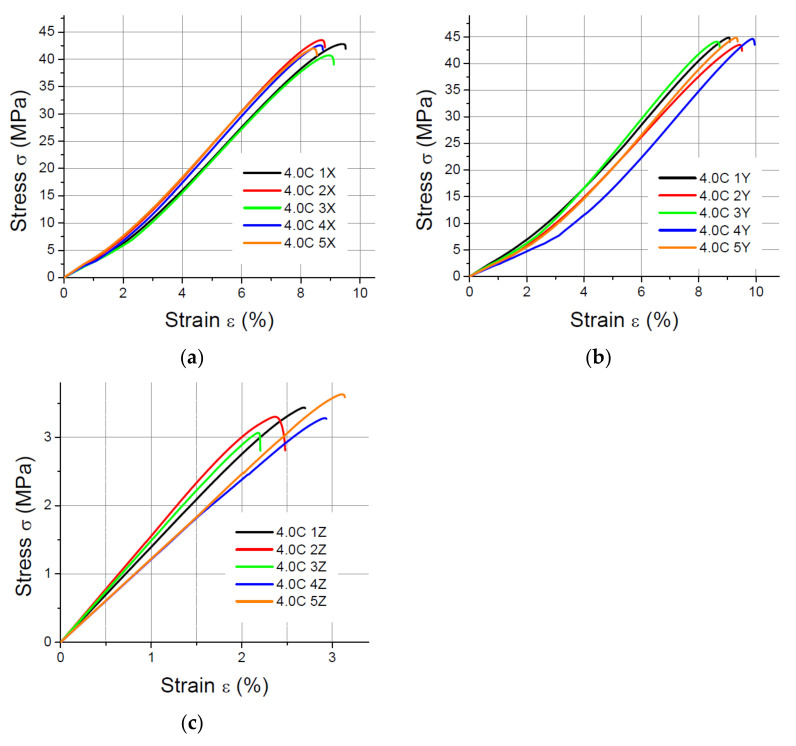
Tensile test results for 4 mm thick specimens made of PLA-CF printed in (**a**) the X orientation; (**b**) the Y orientation; (**c**) the Z orientation.

The results of the static tensile strength tests performer for PLA-CF prints are shown in Figure 8, Figure 9, Figure 10 and Figure 11.

The ultimate tensile strength *R_m_* and the maximum percentage deformation *ɛ_m_* of the PLA and PLA-CF specimens observed at a maximum tensile force are given in Table 4 and Table 5, respectively.

The values of the elasticity modulus E estimated using the regression method from the data of the static tensile strength tests obtained for the PLA and PLA-CF specimens are provided in Table 6 and Table 7, respectively.

### 3.3. Microscopy

**Figure 12:**
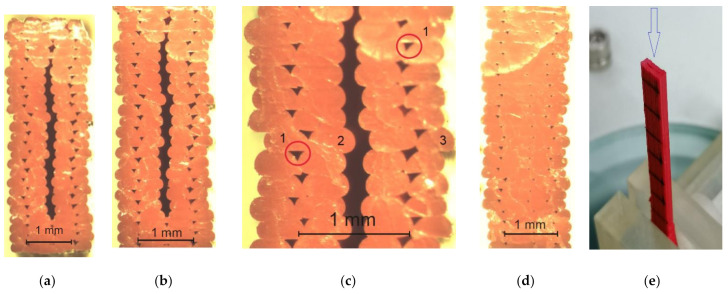
Cross-sectional views of the PLA specimens examined with a stereo microscope; (**a**) a 1.8 mm thick specimen, ×6 magnification, (**b**) a 1.8 mm thick specimen, ×12 magnification, (**c**) a 1.8 mm thick specimen, ×25 magnification, (1—voids at the interface between the neighboring layers, 2—a layer displaced inwards, 3—a layer displaced outwards), (**d**) a 1.4 mm thick specimen, ×12 magnification, (**e**) top view of samples during microscopy measurement.

**Figure 13:**
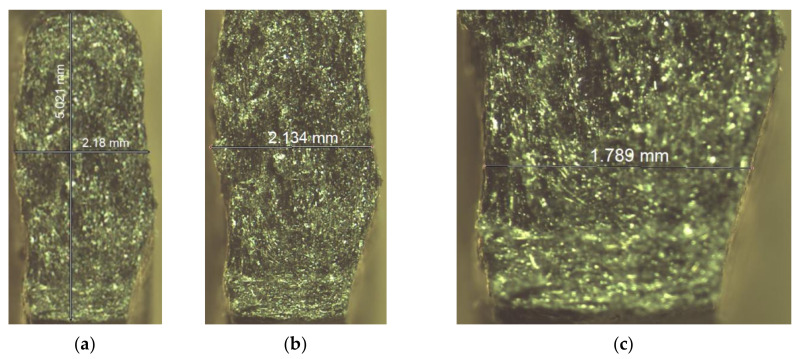
Cross-sectional views of PLA-CF specimens observed after failure using a stereo microscope; (**a**) a 1.8 mm thick Scheme ×6 magnification, (**b**) a 1.8 mm thick specimen, ×12 magnification, (**c**) a 1.8 mm thick specimen, ×25 magnification.

Figure 12 and Figure 13 show images of selected specimens after failure in the fracture area observed using stereo microscopy. Figure 12a–c depicts cross-sectional views of PLA specimens with a thickness of 1.8 mm printed in the Y orientation after failure. As can be clearly seen, there is some delamination of the material (separation of layers) inside the specimens. No similar gaps were present in the other specimens. However, in all the specimens, there are voids in the material (circles marked 1 in Figure 10c). Some layer displacements were also reported. For example, layer 3 was moved outwards or layer 2 was moved inwards.

Figure 13 shows cross-sectional views of 1.8 mm thick PLA-CF specimens printed in the Y direction after tensile testing. As can be seen, the material is clearly deformed. However, the gap in between the layers is less visible than for PLA. Many observations were conducted to select specimens for further examinations with a JEOL JSM-7100F scanning electron microscope.

**Figure 14:**
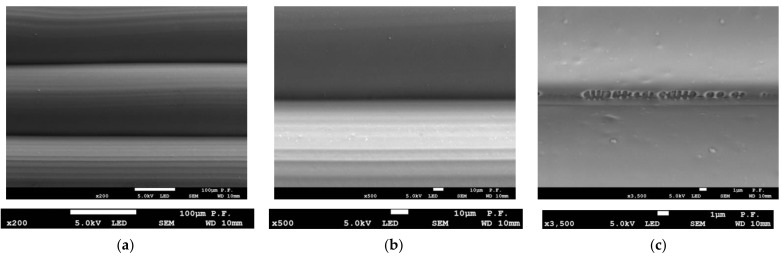
Side views of layers 1.8 mm thick PLA specimen before failure, (**a**) ×100 magnification (**b**) two filaments in contact—×500 magnification, (**c**) inappropriate bonding of two filaments—×3500 magnification.

**Figure 15:**
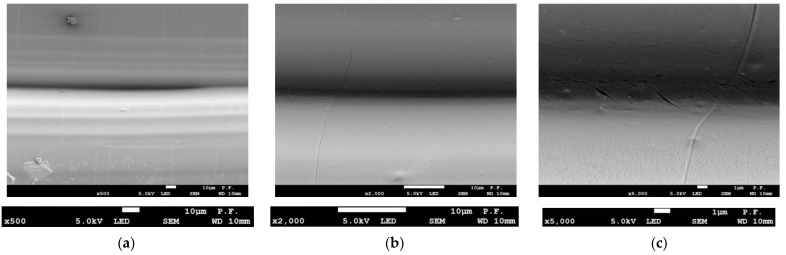
Side views of layers 1.8 mm thick PLA specimen after failure, (**a**) ×500 magnification (**b**) two filaments in contact—×2000 magnification, (**c**) microcracks in the bonding zone between two filaments—×5000 magnification.

**Figure 16:**
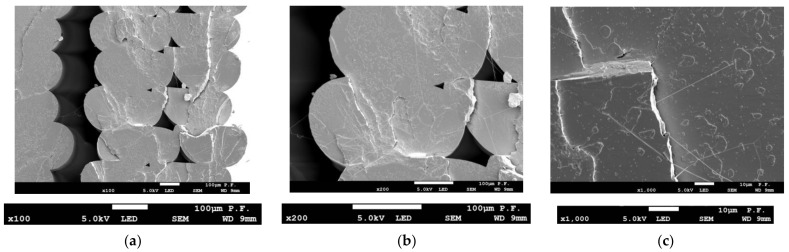
Cross-sectional views of a 1.8 mm thick PLA specimen after failure, (**a**) voids in the material and a gap between the layers, ×100 magnification; (**b**) ×200 magnification, (**c**) microcracks in a single filament, ×5000 magnification.

Scanning electron microscopy was used to examine the flat surface of the specimens before and after tensile tests in the fracture area. Figure 14 shows the flat surface of a 1.8 mm thick PLA specimen printed in the Y orientation at three magnifications. Only at ×3500 magnification can local changes be observed at the interface between the particular layers of the filament placed. Most probably, they are the reason why the layers did not cling to one another and there was a decrease in adhesion. Figure 15 shows a PLA specimen after fracture. In Figure 15a, the ×500 magnification reveals changes at the interface between the filament layers. At a magnification of ×5000 (Figure 15c), there are visible microcracks in the bonding area growing at an angle of 45° to the specimen axis. The direction of cracks coincides with the direction of the maximum tangential stresses. Figure 16 shows voids in the material and a gap between the layers observed with a stereo microscope (the same samples which in Figure 12), magnified ×100 (Figure 16a); displacement of layers, and cracking in the cross-section of a single filament (Figure 16b) at ×5000 magnification.

**Figure 17:**
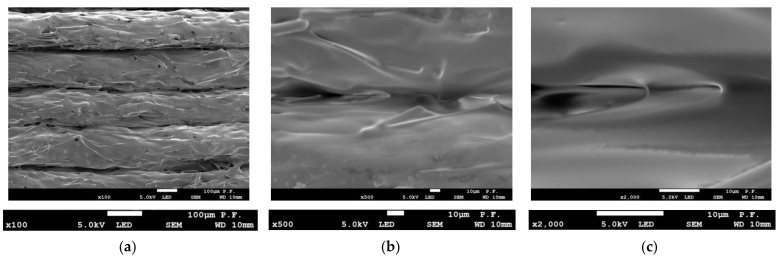
Side views of a 1.8 mm thick PLA-CF specimen before failure, (**a**) ×100 magnification, (**b**) contact zone between two filaments, ×500 magnification, (**c**) abnormalities at the bonding zone of two filaments, ×2000 magnification.

**Figure 18:**
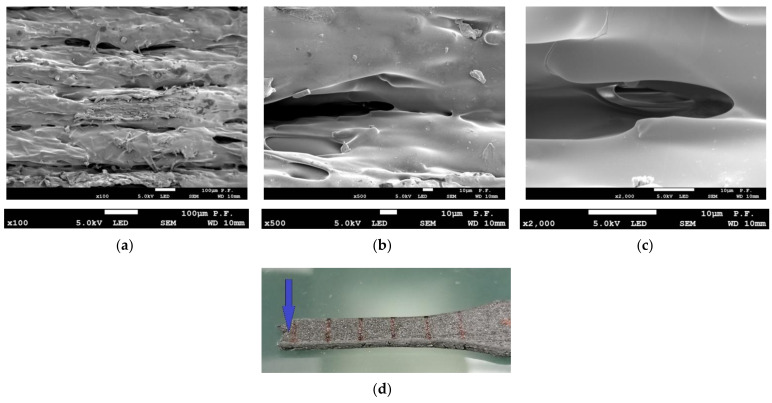
Side views of a 1.8 mm thick PLA-CF specimen after failure, (**a**) ×100 magnification, (**b**) contact zone between two filaments, visible delamination, ×500 magnification, (**c**) delamination at the bonding zone of two filaments, ×2000 magnification, (**d**) view of the tested sample.

Figure 17 shows a flat surface of a 1.8 mm thick PLA-CF specimen printed in the Y orientation at three magnifications. At a magnification of ×100 (Figure 17a), the layers of filament are less regular than those observed for pure PLA, which is due to the presence of the carbon fiber reinforcement. Magnifications ×500 and ×2000 (Figure 17b,c, respectively) reveal abnormalities in the bonding zone between layers of filament. Figure 18 shows a PLA-CF specimen after failure. As can be seen from Figure 18a (×100 magnification), there are changes at the interface between the filaments. At a magnification of ×500 (Figure 18b), however, there is clear delamination of the material in the bonding zone, which is even more visible at a magnification of ×2000 (Figure 18c).

**Figure 19:**
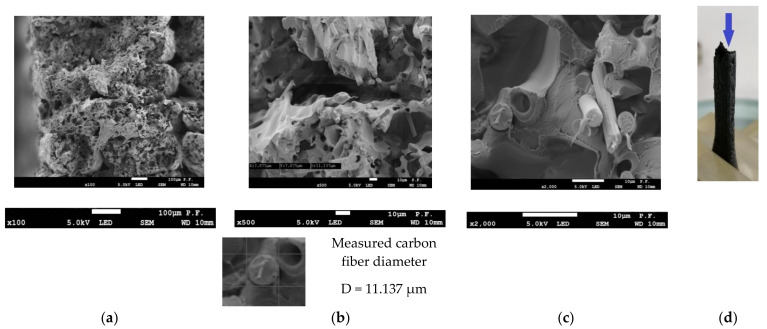
Cross-sectional views of a 1.8 mm thick PLA-CF specimen after failure, (**a**) ×100 magnification, visible voids in the material and a gap between the layers (**b**) ×500 magnification, area of local fracture; cross-sectional view of a carbon fiber (measured diameter) is provided below, (**c**) broken carbon fibers and holes where fibers were, ×2000 magnification, (**d**) view of the tested sample.

**Figure 20:**
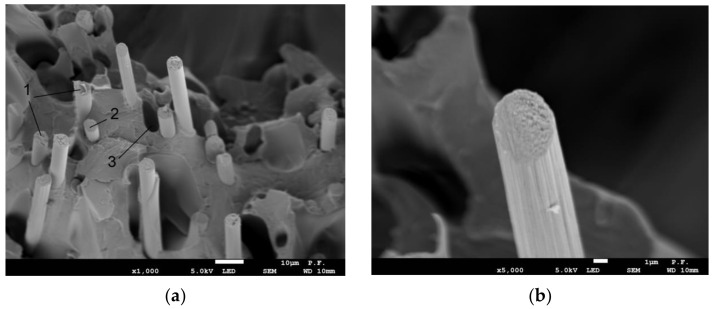
Cross-sectional views of a 1.8 mm thick PLA-CF specimen after fracture (axonometric view), (**a**) ×1000 magnification, 1—broken fibers, 2—non-damaged fibers that partly slid out of the base material, 3—hole where a carbon fiber was, (**b**) ×5000 magnification—a single carbon fiber reinforcing PLA-CF.

Figure 19 and Figure 20 depict the cross-sectional views of the 1.8 mm thick PLA-CF specimens printed in the Y-orientation. The normal view in Figure 19 was taken by the microscope head set perpendicular to the cross-sectional surface. For the axonometric view in Figure 20, the microscope head was placed at an angle to the cross-sectional surface. The ×100 magnification in Figure 19a reveals areas where the material did not bond. The ×500 magnification in Figure 19b shows an area of local failure and a cross-section of a carbon fiber with a measured diameter. Figure 19c illustrates broken carbon fibers and holes where fibers were at a magnification of ×2000.

The axonometric view of a specimen after failure magnified ×1000 in Figure 20 shows broken fibers (1), non-damaged fibers (2) well-embedded in the base material, and holes where carbon fibers were but slid out due to their poor adhesion to the base material (Figure 20a). Figure 20b illustrates a single carbon fiber magnified ×5000.

## 4. Discussion

The experiments had two major objectives: to assess the geometrical accuracy of the specimens and to determine their mechanical properties. Such analysis is essential when 3D objects are printed. The process of printing requires that an element built on the build plate should have not only the predetermined geometry but also the predetermined mechanical properties. Layers of filament are placed successively to form a specific macrostructure, and the bonding of layers is cohesive, adhesive or mixed in nature. The problems considered here are:

− the differences between the nominal (designed) and actual dimensions of the 3D printed thin-walled specimens dependent on their orientation on the build plate (Table 1 and Table 2);
− the influence of the element orientation on the build plate on the ultimate tensile strength *R_m_*, the maximum deformation *ɛ_m_* in percentage (Table 3 and Table 4), and the elasticity modulus E (Table 5 and Table 6).

The best way to assess the measurement data concerning the thickness and width of the specimens is by calculating the relative errors, Δ*a* and Δ*b*, respectively, for each series of measurements. The formula used in the calculations are [20]:

(2) $$\Delta a_{X,Y,Z}=\frac{\left|a-{\bar{a}}_{X,Y,Z}\right|}{a}\cdot 100\%,$$

where: *a*—nominal thickness of the specimen, e.g., *a* = 1.4 mm, ${\bar{a}}_{X,Y,Z}$—average thickness of the specimens in a given measurement series calculated on the basis of the results given in Table 1 or Table 2; for example, for *a* = 1.4 mm (Table 1) and the X orientation, ${\bar{a}}_{X}$ = 1.59 mm; and

(3) $$\Delta b_{X,Y,Z}=\frac{\left|b-{\bar{b}}_{X,Y,Z}\right|}{b}\cdot 100\%,$$

where: *b*—nominal width of the specimen, e.g., *b* = 5 mm, ${\bar{b}}_{X,Y,Z}$—average width of the specimens in a given measurement series calculated on the basis of the results given in Table 1 or Table 2; for instance, for *b* = 5 mm, *a* = 1.4 mm, and the X orientation (Table 1), ${\bar{b}}_{X}$ = 5.37 mm.

**Figure 21:**
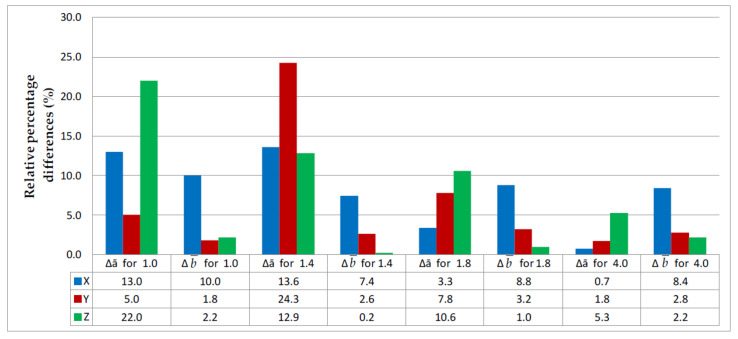
Relative differences in thickness and width between the PLA specimens, where X, Y and Z are printing directions.

**Figure 22:**
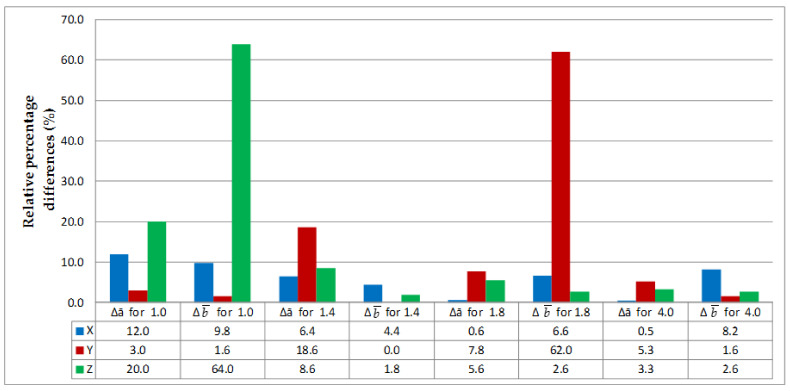
Relative differences in thickness and width between the PLA-CF specimens, where X, Y and Z are printing directions.

The relative errors for thickness (Δ*a*) and width (Δ*b)* were calculated for each series of measurements from the data given in Table 1 and Table 2; the results are represented graphically in Figure 21 and Figure 22.

The largest differences in width between the nominal and actual dimensions were reported for thin-walled specimens made of PLA-CF 1 mm in thickness printed in the Z direction and those 1.8 mm in thickness built in the Y direction, reaching 64% and 62%, respectively. By analyzing the numerical data presented in Figure 21 and Figure 22, it can be clearly stated that in the case of 3D printing of thin-walled models, there are large discrepancies related to the dimensional anisotropy and slicing process of digital model. This phenomenon does not occur on a large scale in the case of samples with a thickness of 4 mm, which confirms the validity of 3D printing studies of thin-walled elements.

**Figure 23:**
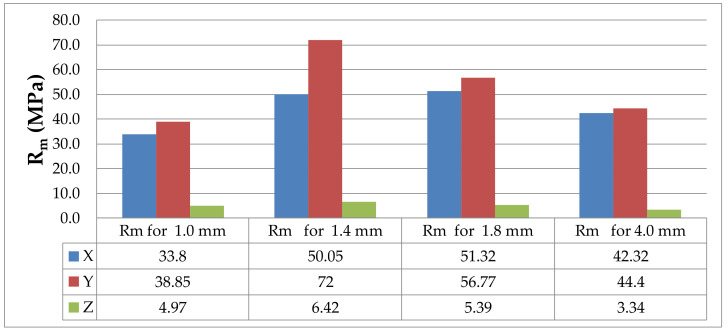
Ultimate tensile strength R_m_ for PLA-CF specimens differing in thickness and print direction, where X, Y and Z are printing directions.

The analysis of the ultimate tensile strength of the particular series of specimens shows that the lowest values were obtained for PLA-CF specimens printed in the Z or vertical direction. The tensile test results are provided in Table 2; and they are represented graphically in the bar chart in Figure 23.

From the bar charts in Figure 22 and Figure 23, it is apparent that the tensile strength of the thin-walled PLA-CF specimens including those 4 mm in thickness was much lower when the printing was undertaken in the Z, i.e., vertical direction. This suggests that the adhesive forces for objects printed in the Z-direction were smaller than for those built in the X- or Y directions. Poor adhesion of carbon fibers added to the base material may also have contributed to that. The microscopic examinations showed that since carbon fibers were parallel to the filament axis, PLA-CF specimens built in the X or, particularly, the Y orientation, had higher strength properties than those made of pure PLA printed in the corresponding orientations. Carbon fiber increased the strength of the base material when the printing was in the X or Y orientation. For instance, 1.8 mm thick PLA specimens built in the Y orientation had R_m_ = 43.05 MPa. For the PLA-CF specimens of the same thickness and orientation, R_m_ was 56.77 MPa, as shown in Table 3.

**Figure 24:**
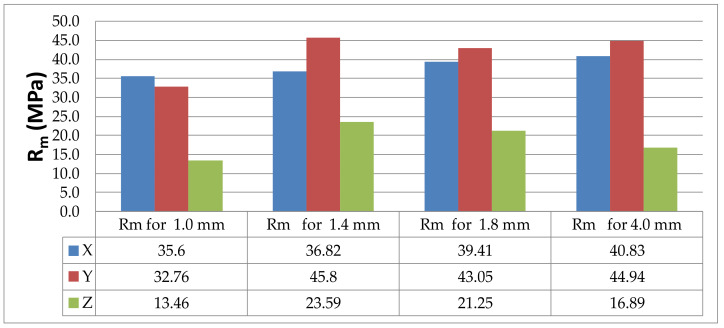
Ultimate tensile strength R_m_ for the PLA specimens differing in thickness and print direction, where X, Y and Z are printing directions.

Similar observations were made for the PLA specimens. The ultimate tensile strength R_m_ was lower for the specimens printed in the Z direction than for those built in the X or Y direction (Table 4 and Figure 11 and Figure 24).

From the considerations so far, it is evident that the tensile strength of FDM prints built in the Z (vertical) direction is lower than that of the other types of specimens. The main forces are the adhesive forces bonding the layers of filament, which are smaller than the cohesive ones. The 1.4 mm thick PLA-CF specimens were reported to have higher strength than the other specimens, which confirms the suitability of this composite to build models characterized by higher mechanical properties.

The investigations, including microscopic observations, revealed voids inside the specimens, as was the case with the PLA specimens 1.8 mm in thickness, and a large gap between the neighboring layers of filament. The microscopic examinations of the PLA-CF specimens showed that carbon fibers could break or slide out of the base material due to the stresses acting on them. Carbon fibers that were well embedded in the base material, i.e., PLA, also transferred tensile stresses, so the specimens printed in the X or Y orientation had higher strength than those built in the Z orientation. The PLA specimens reinforced with CF generally had higher strength than the specimens made of pure PLA. However, carbon fibers reduced the strength of elements printed in the Z orientation because carbon filaments were placed in the direction perpendicular to the tensile stresses. As a result, they did not transfer stresses along their axes; the filaments were weakened in the cross-section. Microscopic studies also showed that carbon fibers were responsible for a greater number of problems in the area of bonding of the filament layers. Similar conclusions were drawn by Valvez et al. [23], who observed that the primary shortcoming of composites was poor adhesion of carbon fiber to the base material. They suggested that future research should focus on solving this problem.

**Figure 25:**
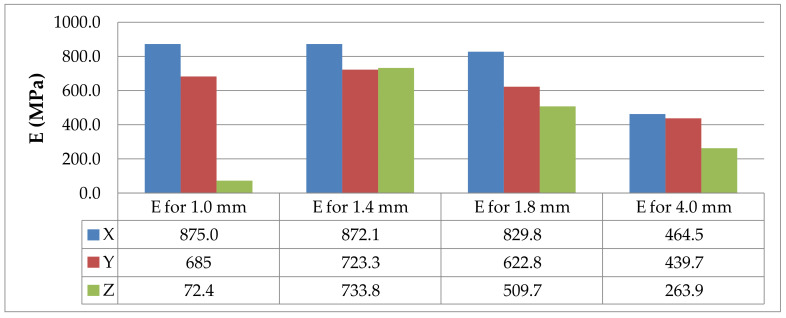
Elasticity modulus E for the PLA specimens differing in thickness and print direction, where X, Y and Z are printing directions.

**Figure 26:**
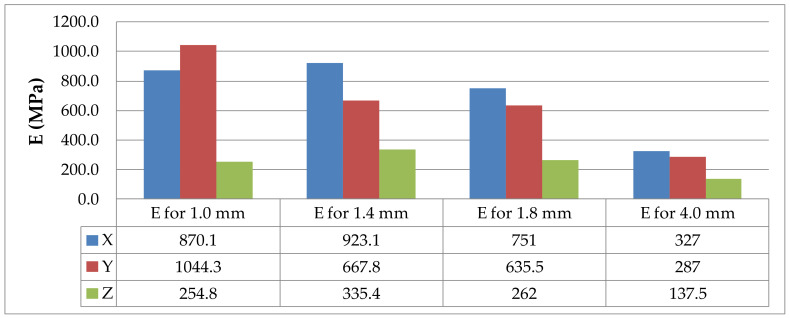
Elasticity modulus E for the PLA-CF specimens differing in thickness and print direction, where X, Y and Z are printing directions.

The tests described here revealed that the elasticity modulus E was dependent on the printing direction, the specimen thickness, and the type of printing material. Generally, the values of the elasticity modulus were lower for the specimens built in the Z direction than for those printed in the X or Y orientation (Figure 25 and Figure 26, respectively); this was due to the arrangement of filaments.

It is interesting to note that whichever material was used (PLA or PLA-CF), the elasticity modulus was lower for the thicker reference specimens (4 mm) than for the thinner ones, as shown in Figure 26. This quantity was reported not to be dependent on the print direction. The bilinear trend of the stress–strain curves may be due to several reasons:

− the result of the specific macrostructural structure of the material produced with the 3D printing technology,
− the result of the behavior of polymers described in terms of rheology by multi-parameter rheological models, e.g., the multi-parameter Maxwell–Wiechert model.

The problem is difficult to interpret unequivocally.

## 5. Conclusions

Summing up the considerations on the mechanical properties of thin-walled PLA and PLA-CF prints built using the FDM technology, we can conclude that like all new engineering materials, these innovative materials showing orthotropic properties should be tested thoroughly considering various aspects of their fabrication. The analysis of the tensile test results and the microscopic data was undertaken to formulate the following conclusions.

1. The FDM technology has a high potential to use different materials. It is possible, for example, to modify the base material by reinforcing it with carbon fiber, which may improve or worsen the mechanical properties of the print, depending on the orientation.
2. The analysis of the mechanical properties of the thin-walled specimens showed that the tensile strength of the thinner specimens (1.0–1.8 mm in thickness) was generally much higher than that of the 4 mm reference specimens. The results can be useful to both design and manufacturing engineers as well as those dealing with the standardization of this 3D printing technology.
3. The base material, PLA, has anisotropic properties; its behavior changes depending on the print orientation. This observation is important because when carbon fiber is used, the composite, i.e., PLA-CF, becomes a typical orthotropic material.
4. The research results presented here can be used in future studies on the FDM printing method to further reduce the number and size of voids and gaps and to modify the base material by adding different components, e.g., carbon fiber.
5. It is vital to properly match the printing parameters to the material used. The printer and the software used in the study were suitable for the purpose.
6. The infill density parameter was found to be responsible not only for the amount of material used on the inside of the object printed but also for the presence of voids and gaps contributing to the material degradation when under tension. Furthermore, there were microckracks in the bonding zone along the direction of the maximum tangential stress, which also led to the material degradation when loaded.
7. The test results show that the elasticity modulus of thin-walled models varies depending on the printing direction and the specimen thickness. This relationship was particularly visible for the thinnest specimens, i.e., those 1 mm in thickness.

## Figures and Tables

**Table 1:** Mechanical properties of polylactic acid (PLA) and polylactide and carbon fiber (PLA-CF) [29].

Properties	PLA, Easy PLA	PLA-CF, CarbonFil
Specific gravity	1.24 g/cm^3^	1.19 g/cm^3^
Tensile modulus	3600 MPa (ASTM D882)	3800 MPa (ASTM D256)
Elongation at break	6% (ASTM D882)	8% (ISO 527)
Print temperature	200–230 °C	230–265 °C

**Table 2:** Dimensions of the PLA prints before the tensile tests.

No.	*ā* (mm)	$\bar{b}$ (mm)	No.	*ā* (mm)	$\bar{b}$ (mm)	No.	*ā* (mm)	$\bar{b}$ (mm)	No.	*ā* (mm)	$\bar{b}$ (mm)
1.0 1X	1.15	5.52	1.4 1X	1.56	5.38	1.8 1X	1.85	5.46	4.0 1X	3.98	5.39
1.0 2X	1.13	5.55	1.4 2X	1.63	5.38	1.8 2X	1.88	5.44	4.0 2X	3.96	5.36
1.0 3X	1.13	5.5	1.4 3X	1.58	5.36	1.8 3X	1.87	5.43	4.0 3X	3.95	5.44
1.0 4X	1.14	5.45	1.4 4X	1.61	5.33	1.8 4X	1.85	5.42	4.0 4X	3.98	5.45
1.0 5X	1.1	5.47	1.4 5X	1.55	5.38	1.8 5X	1.86	5.43	4.0 5X	3.96	5.46
$\bar{x}$	**1.13**	**5.5**	$\bar{x}$	**1.59**	**5.37**	$\bar{x}$	**1.86**	**5.44**	$\bar{x}$	**3.97**	**5.42**
***SD***	**0.02**	**0.04**	***SD***	**0.04**	**0.02**	***SD***	**0.01**	**0.02**	***SD***	**0.01**	**0.04**
1.0 1Y	1.06	5.06	1.4 1Y	1.79	5.16	1.8 1Y	1.99	5.15	4.0 1Y	4.07	5.12
1.0 2Y	1.06	5.11	1.4 2Y	1.71	5.16	1.8 2Y	1.95	5.15	4.0 2Y	4.07	5.14
1.0 3Y	1.07	5.1	1.4 3Y	1.74	5.12	1.8 3Y	1.92	5.16	4.0 3Y	4.08	5.2
1.0 4Y	1.03	5.09	1.4 4Y	1.76	5.11	1.8 4Y	1.92	5.14	4.0 4Y	4.06	5.11
1.0 5Y	1.01	5.1	1.4 5Y	1.71	5.12	1.8 5Y	1.93	5.18	4.0 5Y	4.09	5.12
$\bar{x}$	**1.05**	**5.09**	$\bar{x}$	**1.74**	**5.13**	$\bar{x}$	**1.94**	**5.16**	$\bar{x}$	**4.07**	**5.14**
***SD***	**0.03**	**0.02**	***SD***	**0.04**	**0.02**	***SD***	**0.03**	**0.02**	***SD***	**0.01**	**0.04**
1.0 1Z	1.19	5.13	1.4 1Z	1.66	5.07	1.8 1Z	1.98	5.01	4.0 1Z	4.2	5.24
1.0 2Z	1.22	5.12	1.4 2Z	1.52	5	1.8 2Z	2	5.07	4.0 2Z	4.16	5
1.0 3Z	1.21	5.18	1.4 3Z	1.54	4.99	1.8 3Z	1.96	5.02	4.0 3Z	4.18	5.16
1.0 4Z	1.3	5.07	1.4 4Z	1.56	4.99	1.8 4Z	2.02	5.05	4.0 4Z	4.21	5.05
1.0 5Z	1.19	5.06	1.4 5Z	1.63	5.02	1.8 5Z	2	5.08	4.0 5Z	4.3	5.11
$\bar{x}$	**1.22**	**5.11**	$\bar{x}$	**1.58**	**5.01**	$\bar{x}$	**1.99**	**5.05**	$\bar{x}$	**4.21**	**5.11**
*SD*	**0.02**	**0.05**	*SD*	**0.06**	**0.04**	*SD*	**0.02**	**0.03**	*SD*	**0.02**	**0.09**

**Table 3:** Dimensions of the PLA-CF prints before the tensile tests.

No.	*ā* (mm)	$\bar{b}$ (mm)	No.	*ā* (mm)	$\bar{b}$ (mm)	No.	*ā* (mm)	$\bar{b}$ (mm)	No.	*ā* (mm)	$\bar{b}$ (mm)
1.0C 1X	1.1	5.4	1.4C 1X	1.3	5.2	1.8C 1X	1.88	5.25	4.0C 1X	4.07	5.42
1.0C 2X	1.4	5.48	1.4C 2X	1.33	5.23	1.8C 2X	1.76	5.36	4.0C 2X	4	5.41
1.0C 3X	0.95	5.68	1.4C 3X	1.29	5.23	1.8C 3X	1.79	5.38	4.0C 3X	4.01	5.5
1.0C 4X	1.09	5.57	1.4C 4X	1.33	5.21	1.8C 4X	1.75	5.32	4.0C 4X	4.01	5.33
1.0C 5X	1.04	5.33	1.4C 5X	1.32	5.24	1.8C 5X	1.75	5.35	4.0C 5X	4.02	5.39
$\bar{x}$	**1.12**	**5.49**	$\bar{x}$	**1.31**	**5.22**	$\bar{x}$	**1.79**	**5.33**	$\bar{x}$	**4.02**	**5.41**
***SD***	**0.17**	**0.14**	***SD***	**0.0182**	**0.02**	***SD***	**0.06**	**0.05**	***SD***	**0.03**	**0.06**
1.0C 1Y	0.95	4.93	1.4C 1Y	1.62	5	1.8C 1Y	1.9	5.12	4.0C 1Y	4.21	5.1
1.0C 2Y	0.94	4.96	1.4C 2Y	1.63	4.99	1.8C 2Y	1.9	5.08	4.0C 2Y	4.3	5.07
1.0C 3Y	1	4.91	1.4C 3Y	1.69	5.03	1.8C 3Y	1.88	4.99	4.0C 3Y	4.19	5.06
1.0C 4Y	0.95	4.92	1.4C 4Y	1.65	5.03	1.8C 4Y	1.9	5.03	4.0C 4Y	4.16	5.02
1.0C 5Y	0.99	4.89	1.4C 5Y	1.73	4.97	1.8C 5Y	1.91	5.03	4.0C 5Y	4.17	5.13
$\bar{x}$	**0.97**	**4.92**	$\bar{x}$	**1.66**	**5**	$\bar{x}$	**1.9**	**5.05**	$\bar{x}$	**4.21**	**5.08**
***SD***	**0.03**	**0.03**	***SD***	**0.02**	**0.01**	***SD***	**0.01**	**0.05**	***SD***	**0.03**	**0.04**
1.0C 1Z	1.14	4.86	1.4C 1Z	1.44	4.91	1.8C 2Z	1.94	4.84	4.0C 1Z	4.2	4.9
1.0C 2Z	1.17	4.9	1.4C 2Z	1.57	4.86	1.8C 4Z	1.9	4.87	4.0C 2Z	4.13	4.91
1.0C 3Z	1.28	4.92	1.4C 3Z	1.43	4.97	1.8C 5Z	1.82	4.86	4.0C 3Z	4.07	4.83
1.0C 4Z	1.19	4.83	1.4C 5Z	1.56	4.83	1.8C 6Z	1.98	4.87	4.0C 4Z	4.13	4.91
1.0C 5Z	1.2	4.85	1.4C 6Z	1.59	4.98	1.8C 7Z	1.87	4.91	4.0C 5Z	4.13	4.79
$\bar{x}$	**1.2**	**4.87**	$\bar{x}$	**1.52**	**4.91**	$\bar{x}$	**1.90**	**4.87**	$\bar{x}$	**4.13**	**4.87**
***SD***	**0.05**	**0.037**	***SD***	**0.08**	**0.066**	***SD***	**0.06**	**0.03**	***SD***	**0.05**	**0.06**

**Table 4:** Ultimate tensile strength and the maximum percentage deformation for the PLA specimens.

No.	*R_m_* (MPa)	*ɛ_m_* (%)	No.	*R_m_* (MPa)	*ɛ_m_* (%)	No.	*R_m_* (MPa)	*ɛ_m_* (%)	No.	*R_m_* (MPa)	*ɛ_m_* (%)
1.0 1X	34.2	3.8	1.4 1X	35.33	3.6	1.8 1X	38.66	4.3	4.0 1X	42.23	6.8
1.0 2X	34.87	3.9	1.4 2X	38.98	4.8	1.8 2X	40.31	4.6	4.0 2X	41.04	7.1
1.0 3X	35.93	3.8	1.4 3X	35.2	3.7	1.8 3X	40.43	4.5	4.0 3X	41.04	6.9
1.0 4X	36.23	3.8	1.4 4X	37.76	4.1	1.8 4X	38.3	4.3	4.0 4X	39.9	7.4
1.0 5X	36.62	3.8	1.4 5X	36.82	4.5	1.8 5X	39.34	4.4	4.0 5X	39.92	6.7
$\bar{x}$	**35.57**	**3.8**	$\bar{x}$	36.82	4.1	$\bar{x}$	**39.41**	**4.4**	$\bar{x}$	**40.83**	**7.0**
***SD***	**1.0**	**0.1**	SD	**1.61**	**0.5**	***SD***	**0.96**	**0.1**	***SD***	**0.97**	**0.3**
1.0 1Y	27.02	4.3	1.4 1Y	42.1	4.9	1.8 1Y	41.18	5.4	4.0 1Y	45.88	8.8
1.0 2Y	35.89	4.6	1.4 2Y	47.54	5.6	1.8 2Y	43.45	6.0	4.0 2Y	44.58	7.8
1.0 3Y	26.12	4.2	1.4 3Y	46.31	5.3	1.8 3Y	43.86	5.4	4.0 3Y	45.08	8.3
1.0 4Y	38.63	4.8	1.4 4Y	46.5	5.9	1.8 4Y	43.44	5.3	4.0 4Y	43.93	7.5
1.0 5Y	36.14	4.3	1.4 5Y	46.55	5.3	1.8 5Y	43.34	5.4	4.0 5Y	45.24	7.9
$\bar{x}$	**32.76**	**4.5**	$\bar{x}$	**45.8**	**5.4**	$\bar{x}$	**43.05**	**5.5**	$\bar{x}$	**44.94**	**8.1**
***SD***	**5.76**	**0.3**	SD	**2.12**	**0.4**	***SD***	**1.07**	**0.3**	***SD***	**0.73**	**0.5**
1.0 1Z	12.7	2.4	1.4 1Z	15.55	2.4	1.8 1Z	20.85	4.0	4.0 1Z	16.92	4.5
1.0 2Z	13.49	1.9	1.4 2Z	28.69	3.3	1.8 2Z	21.8	3.6	4.0 2Z	17.45	4.4
1.0 3Z	14.19	2.1	1.4 3Z	25.05	3.1	1.8 3Z	20.22	3.8	4.0 3Z	16.95	4.9
1.0 4Z	14.22	2.3	1.4 4Z	25.36	3.2	1.8 4Z	21.11	3.4	4.0 4Z	17.16	4.3
1.0 5Z	12.72	1.8	1.4 5Z	23.29	2.7	1.8 5Z	22.27	3.6	4.0 5Z	15.98	4.3
$\bar{x}$	**13.46**	**2.1**	$\bar{x}$	**23.59**	**2.9**	$\bar{x}$	**21.25**	**3.7**	$\bar{x}$	**16.89**	**4.5**
***SD***	**0.75**	**0.3**	***SD***	**4.9**	**0.4**	***SD***	**0.8**	**0.3**	***SD***	**0.55**	**0.2**

**Table 5:** Ultimate tensile strength and the maximum percentage deformation for the PLA specimens.

No.	*R_m_* (MPa)	*ɛ_m_* (%)	No.	*R_m_* (MPa)	*ɛ_m_* (%)	No.	*R_m_* (MPa)	*ɛ_m_* (%)	No.	*R_m_* (MPa)	*ɛ_m_* (%)
1.0C 1X	30.27	3.9	1.4C 1X	51.68	5.0	1.8C 1X	50.7	5.6	4.0C 1X	42.8	9.4
1.0C 2X	25.28	3.3	1.4C 2X	43.89	4.2	1.8C 2X	53.88	6.1	4.0C 2X	43.53	8.7
1.0C 3X	40.39	3.9	1.4C 3X	55.41	4.9	1.8C 3X	49.66	5.1	4.0C 3X	40.72	9.0
1.0C 4X	34.06	4.4	1.4C 4X	50.92	5.3	1.8C 4X	53.34	6.1	4.0C 4X	42.57	8.6
1.0C 5X	38.84	3.4	1.4C 5X	50.61	4.8	1.8C 5X	49.04	5.3	4.0C 5X	41.99	8.4
$\bar{x}$	**33.77**	**3.8**	$\bar{x}$	**50.5**	**4.8**	$\bar{x}$	**51.32**	**5.6**	$\bar{x}$	**42.32**	**8.8**
***SD***	**6.2**	**0.4**	SD	**4.17**	**0.4**	***SD***	**2.18**	**0.5**	***SD***	**1.05**	**0.4**
1.0C 1Y	34.52	3.8	1.4C 1Y	73.69	6.8	1.8C 1Y	55.32	6.7	4.0C 1Y	44.89	9.0
1.0C 2Y	39.32	3.9	1.4C 2Y	74.28	7.0	1.8C 2Y	56.54	6.5	4.0C 2Y	43.5	9.4
1.0C 3Y	36.37	4.1	1.4C 3Y	70.8	7.2	1.8C 3Y	58	6.3	4.0C 3Y	44.1	8.6
1.0C 4Y	34.93	2.7	1.4C 4Y	72.27	6.8	1.8C 4Y	59.51	7.1	4.0C 4Y	44.63	9.9
1.0C 5Y	49.13	4.9	1.4C 5Y	68.94	7.5	1.8C 5Y	54.49	6.4	4.0C 5Y	44.86	9.3
$\bar{x}$	**38.85**	**3.9**	$\bar{x}$	**72**	**7.1**	$\bar{x}$	**56.77**	**6.6**	$\bar{x}$	**44.4**	**9.3**
***SD***	**6.05**	**0.8**	SD	**2.18**	**0.3**	***SD***	**2.02**	**0.48**	***SD***	**0.59**	**0.5**
1.0C 1Z	4.87	2.4	1.4C 1Z	6.66	2	1.8C 2Z	5.25	2.2	4.0C 1Z	3.44	2.7
1.0C 2Z	4.65	1.9	1.4C 2Z	6.7	2.3	1.8C 4Z	4.79	1.9	4.0C 2Z	3.3	2.4
1.0C 3Z	4.85	2.0	1.4C 3Z	5.94	2	1.8C 5Z	6.47	2.3	4.0C 3Z	3.07	2.2
1.0C 4Z	5.02	2.2	1.4C 5Z	6.18	1.8	1.8C 6Z	5.09	1.9	4.0C 4Z	3.28	2.9
1.0C 5Z	5.47	2.5	1.4C 6Z	6.65	2.2	1.8C 7Z	5.36	2.5	4.0C 5Z	3.63	3.1
$\bar{x}$	**4.97**	**2.2**	$\bar{x}$	**6.42**	**2.1**	$\bar{x}$	**5.39**	**2.2**	$\bar{x}$	**3.34**	**2.7**
***SD***	**0.31**	**0.2**	***SD***	**0.35**	**0.2**	***SD***	**0.64**	**0.3**	***SD***	**0.21**	**0.4**

**Table 6:** Elasticity modulus (in MPa) for the PLA specimens.

No.	*E*	No.	*E*	No.	*E*	No.	*E*
1.0 1X	829.4	1.4 1X	1062.5	1.8 1X	854.8	4.0 1X	497.6
1.0 2X	866.6	1.4 2X	756.4	1.8 2X	799.8	4.0 2X	437.2
1.0 3X	865.6	1.4 3X	976.2	1.8 3X	804.7	4.0 3X	486.3
1.0 4X	921.3	1.4 4X	876.9	1.8 4X	844.7	4.0 4X	399.2
1.0 5X	897	1.4 5X	688.6	1.8 5X	844.9	4.0 5X	502
$\bar{x}$	**875**	$\bar{x}$	**872.1**	$\bar{x}$	**829.8**	$\bar{x}$	**464.5**
*SD*	**34.9**	SD	**153.4**	***SD***	**25.5**	***SD***	**44.7**
1.0 1Y	574.1	1.4 1Y	751.4	1.8 1Y	659.8	4.0 1Y	358
1.0 2Y	656.9	1.4 2Y	715.8	1.8 2Y	590	4.0 2Y	486.8
1.0 3Y	645.3	1.4 3Y	752.9	1.8 3Y	628.7	4.0 3Y	394
1.0 4Y	723.7	1.4 4Y	648.9	1.8 4Y	641.4	4.0 4Y	517
1.0 5Y	825.1	1.4 5Y	747.5	1.8 5Y	594.1	4.0 5Y	442.8
$\bar{x}$	**685**	$\bar{x}$	**723.3**	$\bar{x}$	**622.8**	$\bar{x}$	**439.7**
***SD***	**94.6**	SD	**44.3**	***SD***	**30.2**	***SD***	**65.1**
1.0 1Z	68.5	1.4 1Z	600.6	1.8 1Z	468.8	4.0 1Z	293.3
1.0 2Z	80.4	1.4 2Z	801.6	1.8 2Z	531	4.0 2Z	260.9
1.0 3Z	73.7	1.4 3Z	791.7	1.8 3Z	452.5	4.0 3Z	215.8
1.0 4Z	66.2	1.4 4Z	690.2	1.8 4Z	555.1	4.0 4Z	279.3
1.0 5Z	73	1.4 5Z	784.8	1.8 5Z	541.3	4.0 5Z	270.4
$\bar{x}$	**72.4**	$\bar{x}$	**733.8**	$\bar{x}$	**509.7**	$\bar{x}$	**263.9**
***SD***	**5.5**	***SD***	**86.9**	***SD***	**45**	***SD***	**29.4**

**Table 7:** Elasticity modulus (in MPa) for the PLA-CF specimens.

No.	*E*	No.	*E*	No.	*E*	No.	*E*
1.0C 1X	723.5	1.4C 1X	944	1.8C 1X	771.5	4.0C 1X	307.9
1.0C 2X	649.8	1.4C 2X	888.3	1.8C 2X	792.3	4.0C 2X	351.6
1.0C 3X	1000.6	1.4C 3X	1053.9	1.8C 3X	822.6	4.0C 3X	289.6
1.0C 4X	841.1	1.4C 4X	770.7	1.8C 4X	651.8	4.0C 4X	316.8
1.0C 5X	1135.3	1.4C 5X	958.6	1.8C 5X	716.9	4.0C 5X	369.2
$\bar{x}$	**870.1**	$\bar{x}$	**923.1**	$\bar{x}$	**751**	$\bar{x}$	**327**
***SD***	**198.9**	**SD**	**104**	***SD***	**67.6**	***SD***	**32.6**
1.0C 1Y	948.1	1.4C 1Y	763.3	1.8C 1Y	591.8	4.0C 1Y	341.6
1.0C 2Y	1029.4	1.4C 2Y	696.5	1.8C 2Y	638.1	4.0C 2Y	277.1
1.0C 3Y	983.6	1.4C 3Y	651.8	1.8C 3Y	701.9	4.0C 3Y	306.3
1.0C 4Y	1251.6	1.4C 4Y	720.3	1.8C 4Y	545.6	4.0C 4Y	235.4
1.0C 5Y	1008.7	1.4C 5Y	507.3	1.8C 5Y	700.2	4.0C 5Y	274.4
$\bar{x}$	**1044.3**	$\bar{x}$	**667.8**	$\bar{x}$	**635.5**	$\bar{x}$	**287**
***SD***	**119.8**	**SD**	**98.4**	***SD***	**68.2**	***SD***	**39.6**
1.0C 1Z	230.2	1.4C 1Z	363.7	1.8C 2Z	240.5	4.0C 1Z	139.6
1.0C 2Z	268.1	1.4C 2Z	315.7	1.8C 4Z	265.1	4.0C 2Z	155.9
1.0C 3Z	257.6	1.4C 3Z	312	1.8C 5Z	292.9	4.0C 3Z	148.7
1.0C 4Z	260.8	1.4C 5Z	365.1	1.8C 6Z	273.7	4.0C 4Z	121.2
1.0C 5Z	257.4	1.4C 6Z	320.7	1.8C 7Z	238	4.0C 5Z	122.2
$\bar{x}$	**254.8**	$\bar{x}$	**335.4**	$\bar{x}$	**262**	$\bar{x}$	**137.5**
***SD***	**14.4**	***SD***	**26.6**	***SD***	**23.1**	***SD***	**15.6**

## Data Availability

The data created in this study are fully depicted in the article.
